# 50μm *ex vivo* MRI reveals distant layer-specific hippocampal atrophy in early Alzheimer’s disease

**DOI:** 10.21203/rs.3.rs-10800434/v1

**Published:** 2026-09-01

**Authors:** Yasmine Salman, Sandra O. Tomé, Nicolas Joudiou, Amanda Annettesdotter, Pulkit Khandelwal, Tevi M. Lawson, Catherine Behets, Benoît Lengelé, Dietmar R. Thal, Paul A Yushkevich, Bernard Gallez, Laura EM Wisse, Bernard J Hanseeuw

**Affiliations:** 1) Louvain Aging Brain Lab, Institute of Neuroscience, UCLouvain, 1200 Brussels, Belgium.; 2) Laboratory of Neuropathology - Department of Imaging and Pathology and Leuven Brain Institute (LBI), KU Leuven (University of Leuven), 3000, Leuven, Belgium.; 3) Nuclear and Electron Spin Technologies (NEST) Platform, Louvain Drug Research Institute, UCLouvain, 1200 Brussels, Belgium.; 4) Department of Clinical Sciences Lund, Lund University, 22185, Lund, Sweden.; 5) Department of Radiology, Massachusetts General Hospital, Harvard Medical School, Boston, MA 02155, USA.; 6) Department of Radiology, University of Pennsylvania, Philadelphia, PA 19103, USA.; 7) Department of Neurosurgery, Cliniques Universitaires Saint-Luc, 1200 Brussels, Belgium.; 8) Morphology Lab (MORF), Institut de recherche expérimentale et clinique (IREC), UCLouvain, 1200 Brussels, Belgium.; 9) Department of Plastic and Reconstructive Surgery, Cliniques Universitaires Saint-Luc, 1200 Brussels, Belgium.; 10) Department of Neuropathology, UZ Leuven, 3000, Leuven, Belgium.; 11) Biomedical Magnetic Resonance Research Group, Louvain Drug Research Institute, UCLouvain, 1200 Brussels, Belgium.; 12) Department of Neurology, Cliniques Universitaires, 1200 Brussels, Belgium.; 13) WELBIO department, WEL Research Institute, 1300 Wavre, Belgium.

**Keywords:** *Ex vivo* MRI, Ultra-high-resolution imaging, Alzheimer’s Disease, Tau pathology, Medial Temporal Lobe

## Abstract

Tau pathology is a major driver of neurodegeneration in Alzheimer’s disease, yet its earliest structural correlates remain poorly understood because of the limited resolution of *in vivo* magnetic resonance imaging (MRI). We studied 19 postmortem brains spanning Braak neurofibillary tangle (NFT) stages I (*N* = 9) and II (*N* = 10) using ultra-high-resolution *ex vivo* MRI of the medial temporal lobe (MTL) at 50 × 50 μm^2^ in-plane resolution and 100 μm slice thickness. MTL subregions and layers were delineated across the anterior and posterior MTL, including layers of cornu ammonis (CA)1 and CA3, subiculum, dentate gyrus, transentorhinal cortex (Brodmann area 35 [BA35]), and entorhinal cortex (ERC). Associations between AT8-positive NFT density and regional thickness were assessed using nonparametric models controlling for demographic factors and co-pathologies. Ultra-high-resolution MRI enabled visualization of previously inaccessible mesoscopic features, including dentate gyrus lamination, subicular boundaries, and the internal organization of the stratum radiatum/lacunosum-moleculare. Compared with Braak stage I, Braak stage II cases showed significantly lower thickness of the anterior CA1 pyramidal cell layer and anterior subicular stratum lacunosum-moleculare, with findings remaining robust across sensitivity analyses. Anterior CA1 and subicular thinning were strongly associated with NFT burden in early Braak-affected regions (ERC/BA35), rather than with local tau pathology, supporting a distant effect of tau on neurodegeneration. Interaction analyses further suggested that the association between tau pathology and CA1 atrophy differed according to TDP-43 status. These findings demonstrate that tau-related atrophy is detectable as early as Braak stage II and preferentially affects anterior CA1 and subiculum at the laminar level, supporting a network-level mechanism of early neurodegeneration.

## 5. Introduction

Alzheimer’s disease (AD) is a slowly progressive neurodegenerative disorder characterized by the accumulation of amyloid-beta (Aβ) plaques and hyperphosphorylated tau neurofibrillary tangles (NFTs), ultimately leading to neurodegeneration, cognitive decline, and dementia [37, 52]. Among these pathological hallmarks, tau pathology shows the strongest association with neuronal loss and cognitive impairment [26, 33, 34, 57].

Tau NFTs show a characteristic pattern of progression, with the medial temporal lobe (MTL), a key region for episodic memory, being among the earliest cortical regions affected. Pathology emerges in the transentorhinal and entorhinal (ERC) cortices at Braak stage I, then involves the hippocampus, particularly cornu ammonis (CA) 1 and subiculum (SUB), at Braak stage II, and subsequently extends to the amygdala and neocortex [52, 4, 11, 13, 14, 16].

Hippocampal subfields are structurally heterogeneous and organized into distinct layers[38]. In the CA fields, these include the alveus (superficial efferent fibers), the stratum oriens (containing basal dendrites and interneurons), the stratum pyramidale (pyramidal cell bodies), and the strata radiatum, lacunosum, and moleculare (largely synaptic layers) [38]. In contrast, the SUB exhibits a simpler laminar organization, typically composed of a superficial molecular layer consisting of ‘the strata lacunosum moleculare’, a principal pyramidal cell layer, and a deep polymorphic layer adjacent to the white matter/angulum bundle [38]. Observing differential atrophy across these laminar organizations would suggest a selective vulnerability of specific neuronal populations to early tau accumulation [11, 81].

Despite the long preclinical phase of AD [67, 68, 79], current *in vivo* approaches fail to capture these earliest changes[82]. Structural MRI of the MTL combined with cerebrospinal fluid [25, 27, 64], tau positron emission tomography [8, 21, 75, 92–94], or plasma biomarkers [30] has been widely used to define early MRI signatures of tau pathology and probe early disease stages. However, *in vivo* biomarkers for tau pathology lack sensitivity to Braak stages I and II and predominantly detect more advanced pathology [50, 78, 82].

Another major challenge lies in the presence of multiple co-occurring proteinopathies within the MTL, including TDP-43 [59, 60, 91] and α-synuclein [29, 31, 43, 83]. This further obscures the identification of tau-specific imaging signatures, as their effects potentially overlap by the time they become detectable *in vivo*.

Attempts to disentangle these contributions using antemortem MRI remain limited by spatial resolution and by the temporal gap between imaging and neuropathological assessment, and have largely focused on later disease stages [7, 20, 23, 24, 32, 35, 39, 42, 44, 46, 54, 76, 84, 88]. In contrast, *ex vivo* MRI enables ultra-high-resolution imaging of the MTL with no temporal delay relative to histopathological examination, allowing the investigation of hippocampal subfields and their laminar organization with unprecedented detail [2, 5, 22, 70, 77, 87]. Although *ex vivo* MRI has shown promise for linking volumetric measures to neuropathology, prior work has mainly focused on advanced tau stages [70, 47, 71, 73, 80, 89, 95]. To our knowledge, only one study has investigated the association between tau pathology and atrophy at early asymptomatic Braak stages, focusing exclusively on the ERC and its subfields [49]. Whether these early structural alterations extend to hippocampal subfields, particularly at the laminar level, remains unknown.

Here, we leverage ultra-high-resolution *ex vivo* MRI acquired at 11.7 tesla (T) to investigate the impact of early tau pathology on granular MTL structural measures. Using a unique cohort of 19 postmortem brains spanning Braak stages I and II, scanned at 50 × 50 μm^2^ in-plane resolution and 100 μm slice thickness, we investigated (i) whether laminar structures of hippocampal subfields can be reliably delineated and support quantitative analyses at this scale, (ii) whether tau-related structural differences are detectable at Braak stage II, and (iii) whether these effects are modulated by co-occurring proteinopathies. By focusing on the earliest stages of tau pathology, this study aims to define the initial structural signature of tau accumulation and to inform the future development of sensitive MRI biomarkers for preclinical Alzheimer’s disease.

## 6. Material and methods

### 6.1. Participants

Brain hemispheres were obtained from 19 donors who underwent intracranial harvest at UCLouvain, Brussels, Belgium. Brain procurements were performed in accordance with Belgian law after approval by the ethical committee of UCLouvain (Date: 1^st^ July 2020, study number UCL-2020–355). All donors provided written informed consent during life through a body or brain donation program (UCLouvain), and additional consent was obtained from next of kin at the time of death. Fourteen donors were cognitively unimpaired and enrolled in the university’s body or brain donation program, while the remaining five were cognitively impaired.

### 6.2. Brain preparation for *ex vivo* MRI

The donor brains were removed 4 to 72 hours after death (see Supplementary Material). Left hemispheres were fixed in 10% neutral buffered formalin for at least 30 days before dissection [74]. After fixation, intact MTL blocks were carefully extracted to preserve amygdalar, hippocampal, and parahippocampal gyrus structures (see Supplementary Material for details). Each MTL specimen was immersed in Fluorinert (3M) [18] within a cylindrical tube that precisely matched the specimen dimensions (diameter × height: 3.5 cm × 7 cm). A custom 3D-printed bubble trap was positioned on top of each specimen, and vacuum was applied to minimize residual air bubbles and thereby reduce MRI susceptibility artifacts [74].

### 6.3. *Ex vivo* MRI acquisition

MTL specimens were scanned on an 11.7T preclinical MRI scanner (Bruker, Biospec) using a 3-D T1-weighted FLASH sequence (TR = 70 ms, TE = 4.425 ms, flip angle = 20°, FOV = 35 × 35 mm^2^). Imaging was performed using a 40 mm inner diameter quadratic birdcage coil. This protocol yielded an in-plane resolution of 0.05 × 0.05 mm^2^ with a slice thickness of 0.10 mm. Total acquisition time was 3.25 hours per specimen.

### 6.4. *Ex vivo* MRI thickness measurements

MRI thickness measurements were performed in two locations in the MTL per specimen: (i) at the level of the anterior hippocampus, defined by the disappearance of the CA3 X-shape (Figure 1A), and (ii) at the level of the lateral geniculate nucleus, at the mid hippocampus (Figure 1B). These locations were defined based on MRI-visible landmarks and therefore represent approximations of the underlying cytoarchitectonic regions, which cannot be fully resolved even at this resolution.

At each location, cortical thickness was sampled at predefined anatomical landmarks (twenty per specimen: twelve anterior and eight posterior; Figure 1C–D). These landmarks were manually placed and used as anchor points for semi-automated segmentation of the surrounding gray matter in ITK-SNAP [96], with manual correction when necessary. For each landmark, a region of interest was defined by extending approximately 5 mm in all directions within the local gray matter. A maximally inscribed sphere is then computed using Voronoi skeletonization [61]. Thickness was then defined as the diameter of the largest sphere centered at each landmark and fully contained within the segmented gray matter [89, 73] (Figure 1E; also see Supplementary Material).

Thickness measurements focused on the pyramidal layer and stratum (radiatum) lacunosum moleculare S(R)LM of SUB, CA1, and CA3, as well as the granular and molecular layers of the dentate gyrus (DG). Here, S(R)LM refers to the thin hypointense “dark band” visible on high-resolution MRI, corresponding mainly to the strata lacunosum and moleculare rather than the stratum radiatum [22] (Figure 1A–B).

Measurements in the anterior location additionally included the ERC and Brodmann area 35 (BA35), corresponding to the transentorhinal cortex, as well as the alveus of CA1 and CA3 (Figure 1A). In the posterior location, the same hippocampal layers were assessed, whereas it was unclear which parahippocampal gyrus subregions were present at this level. The parahippocampal cortex (PHC) was therefore not delineated at this level. CA2, although of interest, could not be reliably identified on MRI and was therefore excluded. Similarly, CA4 and the presubiculum islands, while anatomically distinguishable (see Figure 2G for a visual enlargement, but also visible on Figure 1A–B), were not suitable for thickness measurements and were not analyzed. Thickness measurements were not possible for all regions in every specimen due to tissue damage (e.g., tears) or insufficient contrast (see Supplementary Table 1 for the number of specimens analyzed per region).

### 6.5. Neuropathology

MTL specimens were cut into 0.5 cm slices using a cutting device equipped with a bottom mirror, allowing alignment of the cutting plane with MRI orientation. For the anterior location, the histological slice corresponding to the MRI-defined level was selected, whereas for the posterior location, the MRI slice matching the histological section was identified.

Specimens were then embedded in paraffin for neuropathological assessments. Several neuropathologic parameters were assessed. Phases of Aβ plaque deposition according to Thal et *al*. (2002) were assessed as described previously, based on anti-Aβ17–24 stained brain sections (clone 4G8, BioLegend, USA) [83]. Braak NFT stages were determined as previously described based on anti-pTau202/205 stained sections (clone AT8, ThermoFisher Scientific, USA) [3, 12]. Neuritic plaque scores were assessed according to the Consortium to Establish a Registry for Alzheimer’s Disease (CERAD) guidelines [53]. Limbic age-related TDP-43 encephalopathy neuropathological change (LATE-NC) was diagnosed and staged according to the published guidelines from a consensus working group with a phosphorylated TDP-43 (pTDP-43) antibody (S409/S410, 1:5000, CAC-TIP-PTD-P07, Cosmo Bio, Japan) [60]. Diagnosis and stages of α-synuclein pathology (= Braak Lewy body disease [LBD] stages) were determined according to Braak et *al*. (2003) with anti-aggregated α-synuclein antibody (1:2000, clone 5G4, #MABN389, Merck, Germany) [17]. Argyrophilic grain disease (AGD) and age-related tau astrogliopathy (ARTAG) were also diagnosed according to diagnostic criteria [15, 48]. Additionally, frontotemporal lobar degeneration with TDP-43 pathology (FTLD-TDP) was diagnosed according to established guidelines [51].

5 μm sections of the anterior and posterior MTL underwent immunohistochemistry. Briefly, sections were deparaffinized and processed with citrate buffer for epitope retrieval (pH = 6) for 10 min. A 3-minute formic acid treatment was applied to sections undergoing Aβ_17–24_ and α-synuclein immunostaining. Endogenous peroxidase was blocked in all sections for 5 min in order to avoid unspecific reactions. Sections were incubated overnight with the primary antibodies mentioned above, followed by a secondary anti-rabbit, anti-mouse, horseradish peroxidase antibody incubation for 30 min. 3,3’-diaminobenzidine was used as a chromogen to yield brown reaction products. Counterstaining was performed with hematoxylin.

For the histological quantification of pTau and pTDP-43 pathology, three representative 200× micrographs with a fixed area were acquired from AT8- and pTDP-43-stained sections using a LEICA DM2000 LED microscope (Leica Biosystems, Germany) in the following regions of interest: the granular layer of the DG, the pyramidal layers of the CA3, CA1, and SUB, layer II of the ERC, and layers II/III of BA35. In posterior sections, the ERC location sometimes corresponded to the remaining ERC and sometimes to the PHC; therefore, posterior ERC quantifications of pTau and pTDP-43 were considered as the ERC/PHC region. Images were opened and visually inspected with Fiji/ImageJ software. pTau pretangles and NFTs were manually counted using pTau (AT8)-stained sections. Finally, pTDP-43-positive neuronal cytoplasmic inclusions (including granular, NFT-like, small, large, and round) were also manually counted. In all instances, the multipoint function was used, and counts were represented in density/mm^2^.

Histological quantifications were then averaged by regions corresponding to Braak NFTs stages region-of-interest, with ERC/BA35 (Braak I), CA1/SUB (Braak II), and CA3/DG (Braak ≥ III) to reduce the number of statistical tests. Averages were computed separately for anterior and posterior MTL.

### 6.6. Statistics

Given the relatively small sample size (*N* = 19), non-parametric methods were used for all analyses. Demographic and pathological characteristics were compared between Braak stage groups (I vs II). Continuous variables (age at death, postmortem delay, Aβ phase, CERAD score) were analyzed using Wilcoxon rank-sum tests, and dichotomous variables (sex, presence of co-pathologies including α-synuclein, TDP-43, ARTAG, AGD, and cognitive impairment) using Fisher’s exact tests.

Group differences in regional tau pathology (NFTs and pretangles) between Braak stages I and II were assessed using rank-based ANOVA models. Paired Wilcoxon signed-rank tests, stratified by Braak stage, were used to compare anterior and posterior MTL measurements within each region. Cortical thickness was compared using rank-based ANCOVA between Braak stage I and II, and adjustments for age, sex, and presence of co-pathologies (α-synuclein, TDP-43, ARTAG; coded as binary variables) were made. Results are reported as rank-based regression coefficients (*β_rank*), p-values, and raw mean differences (*Δ*). The models were repeated after excluding individuals with each co-pathology, using separate models for each condition.

Associations between cortical thickness and tau pathology were evaluated using partial Spearman correlations, controlling for age, sex, and presence of co-pathologies (α-synuclein, TDP-43, ARTAG; coded as binary variables). To specifically assess the influence of TDP-43, two complementary approaches were used: (i) exclusion of TDP-43–positive individuals, and (ii) adjustment for quantitative TDP-43-inclusion burden rather than its binary presence. Interaction effects between tau and TDP-43 were evaluated using a non-parametric approach by computing a tau-NFT burden × TDP-43-inclusion burden interaction term and testing its association with cortical thickness in partial Spearman correlation models, adjusted for age, sex, and presence of co-pathologies.

All analyses were performed in R (version 4.3.1) using the *Rfit* and *ppcor* packages. No correction for multiple comparisons was applied due to the small size of this unique dataset.

## 7. Results

### 7.1. High-resolution *ex vivo* MRI reveals mesoscopic anatomical details.

Visual comparison between histological sections (Figure 2A–B) and *ex vivo* MRI (Figure 2C–D) showed that 50 μm resolution enables the visualization of mesoscopic anatomical features not previously described in the MRI literature. At this resolution, hippocampal lamination could be clearly resolved. The dentate gyrus granule cell layer (DG GCL) appeared as a thin hyperintense band (~150–250 μm), while the molecular layer (ML) was identifiable as an adjacent layer with lower signal intensity than the GCL but higher than the SRLM (Figure 2E).

The SRLM of CA1 displayed clear internal organization. Visible sublayers appeared as hypointense bands, contributing to laminar contrast within CA1 (Figure 2F). In contrast, the subicular SLM appeared thicker and showed less evidence of lamination, likely reflecting the absence of the stratum radiatum in SUB. This highlights a marked regional difference in laminar architecture. At a similar location, a sharp transition between CA1 and SUB was observed within the pyramidal cell layer, characterized in MRI by an oblique change in contrast (Figure 2, red dashed lines), consistent with histological differences in neuronal density [63, 72] and potential increases in crossing fibers. Within the subicular complex, presubiculum islands were also resolved, appearing as hypointense triangular structures protruding into the SLM (Figure 2G).

Beyond hippocampus, transitions between the subicular complex and adjacent cortices (e.g., ERC and PHC) were delineated by coordinated changes in cortical thickness and signal intensity, reflecting underlying cytoarchitectural reorganization. Finally, CA3 was clearly identifiable as a distinct band emerging from CA4 and forming an organized laminar structure, particularly in posterior sections (Figure 2H).

Overall, these findings demonstrate that 50 μm *ex vivo* MRI can resolve fine laminar organization and mesoscale anatomical features that remain inaccessible at clinical MRI resolutions (≤7T).

### 7.2. Demographics

Demographic data are presented in Table 1. 47% of the cases were classified as Braak NFT stage I (*N* = 9), and 53% stage II (*N* = 10). The Braak I group was significantly younger at death than the Braak II group (*Δ* = 8.1 years; *P* < 0.05), whereas sex distribution and cognitive status did not differ significantly (*P* = 0.35 and *P* = 0.99, respectively).

Amyloid-β pathology (Aβ phase, CERAD) and other neuropathologies (TDP-43, α-synuclein, AGD) did not differ between groups, except for ARTAG, which was more frequent in Braak II (*P* = 0.03). Postmortem delay was comparable across groups. Detailed information is provided in Supplementary Table 2.

### 7.3. Tau pathology increases from Braak stage I to II across MTL regions

Braak stage II individuals showed higher levels of both NFTs and pretangles across multiple MTL regions compared to stage I (Figure 3), consistent with the expected progression of tau pathology. NFT burden was significantly increased in CA1/SUB subfields in Braak stage II, both anteriorly (*P* < 0.05; *β_rank* = 4.78; *Δ* = 8.22) and posteriorly (*P* < 0.05; *β_rank* = 7.74; *Δ* = 7.67). Increased NFT burden was also observed in early tau regions (ERC/BA35), indicating continued accumulation despite spreading to downstream areas. However, the number of NFT in later tau regions (CA3/DG) did not differ between groups.

Similarly, pretangle burden was higher in Braak stage II in ERC/BA35 (*P* < 0.05; *β_rank* = 7.14; *Δ* = 7.04), anterior CA1/SUB (*P* < 0.01; *β_rank* = 5.36; *Δ* = 6.64), and ERC/PHC (*P* < 0.01; *β_rank* = 3.57; *Δ* = 2.31). A trend was also observed in anterior CA3/DG (*P* = 0.07; *β_rank* = 1.79; *Δ* = 3.70), suggesting further spread of earlier markers of tau pathology to additional hippocampal subfields, yet to a lower extent.

### 7.4. Braak stage II is associated with atrophy in the anterior CA1 pyramidal cell layer

#### 7.4.1. Regional thickness differences across Braak stages

Comparisons of MTL structural measures across Braak stages revealed significant reductions in thickness in a few regions in Braak stage II relative to stage I (Figure 4). Variance inflation factors (all VIFs < 3) indicated no evidence of problematic multicollinearity among the predictors included in the model. The most prominent effect was observed in the anterior CA1 pyramidal cell layer (*P* < 0.001; *β_rank* = −0.21; *Δ* = −0.26 mm). Additional reductions were found in the anterior SUB SLM (*P* < 0.05; *β_rank* = −0.28; *Δ* = −0.22 mm) and posterior CA1 SRLM (*P* < 0.05; *β_rank* = −0.37; *Δ* = −0.22 mm), while the anterior SUB pyramidal layer showed a trend towards thinning (*P* =0.08; *β_rank* = 0.24; *Δ* = −0.15 mm).

#### 7.4.2. Sensitivity analyses

Sensitivity analyses excluding individuals with co-pathology in separate models for each excluded co-pathology consistently showed that individuals with Braak stage I and II only significantly differed in the anterior CA1 pyramidal cell layer thickness (Figure 5). Specifically, when excluding TDP-43–positive individuals (Braak I: *N* = 7; Braak II: *N* = 5), only the anterior CA1 pyramidal cell layer remained significantly reduced in Braak stage II cases (*P* < 0.05; *β_rank* = −0.16; *Δ* = −0.34 mm). Similarly, exclusion of α-synuclein–positive (Braak I: *N* = 7; Braak II: *N* = 5; *P* < 0.05; *β_rank* = −0.18; *Δ* = −0.25 mm) or ARTAG-positive individuals (Braak I: *N* = 9; Braak II: *N* = 5; *P* < 0.01; *β_rank* = −0.21; *Δ* = −0.10 mm) yielded consistent results. Excluding individuals with co-pathologies led to non-significant differences between Braak stage I and II for anterior SUB SLM (Figure 5B), posterior CA1 SLM (Figure 5C), and anterior SUB ML (Figure 5D). Together, these findings identify anterior CA1 pyramidal cell layer thinning as the most robust structural alteration associated with Braak stage II.

### 7.5. Thinner anterior CA1 and SUB thickness is associated with a higher tau NFTs burden in early Braak regions

#### 7.5.1. Associations between anterior CA1/SUB thickness and tau burden across regions

We next aimed to correlate thickness measures with the density of NFTs and pretangles. Partial Spearman correlations showed that NFT burden in early tau regions (ERC/BA35) was significantly associated only with anterior CA1 pyramidal layer (*R* = −0.70, *P* < 0.05) and anterior SUB SLM (*R* = −0.72, *P* < 0.01; Figure 6, upper panels). In contrast, no significant association was observed between MTL structural measures and local NFT burden in anterior CA1/SUB (Figure 6 – lower panels). However, pretangle burden in anterior CA1/SUB showed (trend-level) association with thickness in anterior CA1 pyramidal cell layer (*R* = −0.53, *P* =0.08) and anterior SUB SLM (*R* = −0.60, *P* < 0.05; Supplementary Figure 2). Because CA3/DG exhibited minimal pathology (see Figure 3), consistent with the early Braak stages of the cohort (I–II), in which pathology typically does not yet involve CA3/DG, correlations for this region were not performed. Full Spearman correlation results for all regions are reported in Supplementary Table 4.

#### 7.5.2. Evidence for a distant effect of ERC/BA35 tau pathology

To further test whether the association between ERC/BA35 NFTs and the thickness of the anterior CA1 pyramidal layer and SUB SLM reflected a distant effect of tau pathology, we adjusted correlations for local NFT burden in CA1/SUB. The associations remained significant for the anterior CA1 pyramidal layer (*R* = −0.71, *P* < 0.01, Figure 7A) and at a trend level for SUB SLM (*R* = −0.53, *P* =0.08, Figure 7B).

To determine whether these associations were specifically related to aggregated tau pathology (i.e., NFTs) rather than to earlier tau pathology, we further adjusted the correlations for local pretangle burden in CA1/SUB. Following adjustment, associations between ERC/BA35 NFT burden and thickness were attenuated but remained relatively strong and marginally significant for both the anterior CA1 pyramidal layer (*R* = −0.54, *P* =0.08, Figure 7A) and SUB SLM (*R* = −0.51, *P* =0.08, Figure 7B), suggesting that the relationship was not explained by local pretangle pathology. Although these associations no longer reached statistical significance, correlation coefficients remained relatively preserved. In contrast, associations between local pretangle burden and thickness were markedly reduced after adjustment for ERC/BA35 NFT burden, with correlation coefficients decreasing to R = 0.07 for the anterior CA1 pyramidal layer and to *R* = 0.04 for the SUB SLM (both *P* > 0.80).

Together, these findings indicate that thinning in CA1/SUB is more strongly associated with upstream NFT pathology in ERC/BA35 than with local aggregated or soluble tau pathology within CA1/SUB. These results support the presence of a distant effect of early (trans)entorhinal tau deposition on hippocampal atrophy.

### 7.6. Impact of TDP-43 on early tau-associated atrophy patterns

Given the known contribution of TDP-43 pathology to MTL atrophy [22, 29, 59, 60, 71, 89, 91], we performed exploratory analyses to investigate its potential impact on the MRI atrophy pattern observed in the present study. These analyses should be interpreted with caution, given the limited number of TDP-43-positive individuals (*N* = 7 [*N*_*FTLD*_ = 2; *N*_*LATE-NC*_ = 5], including 4 with TDP-43 inclusions in the hippocampus) and the heterogeneity of TDP-43 pathology, including LATE-NC, FTLD-B, and FTLD-C.

Partial Spearman correlations revealed that the density of TDP-43-positive neurons in the anterior CA1/SUB region was associated with reduced thickness of the CA1 pyramidal cell layer in both the anterior (*R* = −0.58, *P* =0.068) and posterior (*R* = −0.80, *P* < 0.001) MTL. In contrast, no significant associations were observed with the CA1 SRLM or with either the pyramidal or SLM layers of the subiculum (all p > 0.10).

Given the known interaction between tau and TDP-43 pathology [85], additional analyses were performed excluding TDP-43–positive individuals. In this restricted sample, when only FTLD-TDP cases (*n* = 2) were excluded, the associations remained significant for anterior CA1 thickness (*R* = −0.66, *P* < 0.05) and anterior SUB SLM (*R* = −0.84, *P* < 0.01). When both FTLD-TDP and LATE-NC cases were excluded (*n* = 5), the association with anterior CA1 thickness was attenuated (*R* = −0.62), whereas the association with anterior SUB SLM remained strong (*R* = −0.94). These findings suggest that the association between tau pathology and SUB SLM thickness may be less influenced by TDP-43 pathology than the association observed in the anterior CA1 pyramidal layer, although interpretation is limited by the small sample size.

Finally, we investigated whether TDP-43 modifies the effect of tau pathology on cortical thickness using interaction models restricted to the regions associated with NFTs (i.e., anterior CA1 pyramidal layer and anterior SUB SLM). A significant tau × TDP-43 interaction was observed only for the anterior CA1 pyramidal layer with ERC/BA35 NFT burden (*interaction coefficient* = −0.59, *P* < 0.05), indicating that the association between tau pathology and cortical thinning became stronger with increasing TDP-43-inclusion burden. Similar results were obtained after excluding FTLD-TDP cases (*interaction coefficient* = −0.61, *P* =0.06), suggesting that this interaction was not driven solely by FTLD-TDP pathology.

## 8. Discussion

This study aimed to leverage ultra-high-resolution *ex vivo* MRI to identify MTL structures that undergo early tau-related atrophy. Specifically, we used a unique dataset of 19 postmortem brains scanned at 50 × 50 μm^2^ in-plane resolution to investigate both the fine-grained anatomical features resolvable at this spatial scale and the presence of tau-related atrophy at very early Braak NFT stages.

### 8.1. Early tau-related atrophy in anterior CA1 and subiculum

The main objective of this study was to determine whether MTL subregions exhibit atrophy as early as Braak stage II. We observed significant atrophy in Braak stage II, consistent with a previous report [49], suggesting that structural changes can already be detected at the earliest stages of Alzheimer’s disease tau pathology.

Although thickness differences were observed in both anterior and posterior MTL regions, only anterior structures showed significant correlations with NFT density. This observation indicates that atrophy is more closely associated with tau burden in anterior regions, in line with known gradients of tau pathology showing greater tau accumulation and neurodegeneration in the anterior MTL in early Alzheimer’s disease [70, 80, 95]. More specifically, we identified the anterior CA1 pyramidal cell layer and the anterior SUB SLM as regions showing reduced thickness at Braak stage II and significant associations with NFTs in early Braak regions (ERC/BA35). These findings are consistent with the progression of tau pathology, affecting CA1 and SUB at Braak stage II [13, 14, 55] as well as previous postmortem imaging studies showing atrophy in these regions, but at later stages of the disease [70, 71, 73, 89, 10].

Notably, these results indicate that structural atrophy emerges in close spatial and temporal proximity to tau aggregation, in contrast to delays previously reported [68]. This discrepancy may reflect the limited sensitivity of *in vivo* volumetric imaging approaches, which currently lack the spatial resolution needed to detect early, localized changes. Importantly, the atrophy observed in the anterior CA1 pyramidal layer appeared particularly robust, as it remained significant across all sensitivity analyses. In contrast, although anterior subicular SLM thinning was not consistently detected in Braak stage sensitivity analyses after exclusion of co-pathologies, its association with regional tau burden remained strong after accounting for TDP-43 pathology, suggesting that SUB SLM alterations may be more closely linked to tau pathology itself. Interestingly, this pattern differs from the TDP-43 sensitivity analyses, in which associations between tau burden and thickness remained stronger for the SUB SLM than for the anterior CA1 pyramidal layer. This apparent discrepancy likely reflects the different biological questions addressed by these approaches. Whereas our Braak stage analyses assess the ability of a structural measure to discriminate between stages of tau spread, the correlation analyses evaluate its relationship with the continuous burden of tau pathology. Together, these findings suggest that anterior CA1 pyramidal layer thinning may represent the most robust marker of Braak stage progression, whereas SUB SLM alterations may more closely track tau pathology itself. Although several co-pathologies were present in this cohort, the persistence of anterior CA1 thinning across multiple sensitivity analyses suggests that this finding is not solely driven by these concomitant pathologies. Nevertheless, larger cohorts will be required to determine the independent contribution of each proteinopathy to the observed MRI pattern.

We did not observe significant atrophy in BA35 or the ERC when comparing Braak stages I and II, despite tau aggregation in these regions. While this appears inconsistent with previous findings showing ERC/BA35 atrophy [70, 47, 71, 73, 89, 49, 10], it may be primarily driven by methodological factors. In particular, BA35 and ERC are not homogeneously affected by tau pathology: early tau deposition preferentially targets the medial transentorhinal cortex [13, 14] and the lateral ERC [70, 95, 49, 62]. Moreover, tau pathology is known to affect specific cortical layers within these regions [13, 14], which could not be individually resolved or selectively measured in our MRI-based approach. Therefore, it is possible that our thickness measurements did not fully capture the most vulnerable subregions, potentially underestimating early cortical atrophy.

### 8.2. Evidence for network-level effects of early tau pathology on neurodegeneration

Interestingly, we did not observe thickness reduction in the early Braak regions, but rather in regions anatomically connected to them. Moreover, our results suggest that CA1/SUB atrophy might be driven by pathology in the ERC/BA35 regions, indicating a distant effect of tau pathology. Importantly, we also tested whether this effect could be explained by earlier local tau pathology, as indexed by pretangle burden. Associations between ERC/BA35 NFT burden and CA1/SUB thickness persisted after adjustment for local pretangle burden, whereas trending associations between local pretangles and thickness disappeared after adjustment for ERC/BA35 NFT burden. These findings indicate that hippocampal thinning was more strongly associated with upstream NFT pathology in ERC/BA35 than with local pretangle pathology, suggesting that the observed neurodegeneration is not merely a consequence of local pretangle accumulation but rather reflects the influence of aggregated tau pathology arising in ERC/BA35.

This pattern has been widely reported in the literature using *in vivo* MRI, where regions connected to early tau-affected areas exhibit early structural alterations [1, 6, 9, 28, 36, 40]. These findings support the hypothesis of a trans-synaptic mechanism of tau-related neurotoxicity [19, 69], occurring before NFTs formation.

More specifically, our findings are consistent with a retrograde pattern of network degeneration [69]. The ERC projects to CA1, CA3, and SUB, whereas only CA1 and SUB send reciprocal projections back to the ERC [38]. Under a purely anterograde model, early involvement of CA3 might also be expected. However, we observed stronger atrophy and pathological associations in CA1 and SUB than in CA3, a pattern that is compatible with preferential involvement of retrograde pathways [38]. Nevertheless, other early-affected regions may also contribute to this pattern. In particular, the basolateral amygdala, which is affected early by tau, TDP-43, and α-synuclein pathology [13, 17, 56, 59], projects to both CA1 and the subiculum [66, 65] and therefore represents another plausible source of early pathology-related degeneration. Consequently, our findings do not allow us to distinguish between these potential mechanisms. Future studies combining ultra-high-resolution imaging with connectivity approaches could help identify which specific pathways are affected at these very early stages and provide more direct evidence regarding the directionality of tau spread.

### 8.3. Evidence for an interaction between TDP-43 and tau in CA1 atrophy

Given the growing recognition of TDP-43 as an important contributor to MTL atrophy [22, 29, 59, 60, 71, 89, 91], we performed exploratory analyses to assess its potential influence on the MRI changes observed in this study. Quantitative TDP-43-inclusion burden was associated with thinning of the CA1 pyramidal layer, whereas no significant associations were observed for the CA1 SRLM or the subicular layers. Furthermore, the association between tau pathology and anterior SUB SLM thickness remained robust after accounting for TDP-43 pathology and after excluding TDP-43-positive individuals, whereas the association with anterior CA1 pyramidal layer thickness became attenuated. Together, these findings suggest that anterior SUB SLM thinning may more specifically reflect tau-related neurodegeneration, whereas anterior CA1 pyramidal layer thinning appears to result from the combined influence of tau and TDP-43 pathology. This interpretation is consistent with previous neuropathological studies showing that TDP-43 preferentially affects pyramidal neurons in the CA1 and subiculum [70].

We also observed a significant interaction between tau and TDP-43 in the anterior CA1 pyramidal layer, indicating that the association between tau pathology and cortical thinning became stronger with increasing TDP-43-inclusion burden. This observation is in line with previous reports suggesting synergistic effects between tau and TDP-43 pathology [85, 58, 41, 90]. Importantly, the interaction persisted after exclusion of FTLD-TDP cases, suggesting that it is unlikely to be driven solely by FTLD-TDP pathology and may instead reflect the contribution of LATE-NC.

These findings should nevertheless be interpreted cautiously, given the limited number of TDP-43-positive individuals and the heterogeneity of TDP-43 pathologies represented. Confirmation in larger cohorts with sufficient representation of the different TDP-43 subtypes will therefore be required.

### 8.4. Ultra-high-resolution MRI reveals dentate gyrus lamination and subicular boundaries

Although we did not observe a tau effect in the DG and SUB boundaries, *ex vivo* MRI at 50 μm in-plane resolution allows the direct visualization of MTL features that were previously not accessible with lower resolutions. This ultra-high resolution enables the identification of finer anatomical features including the distinct layers of the DG [77], presubicular islands, and the transition between CA1 and SUB, which, to our knowledge, have not previously been described using *ex vivo* MRI. This increased level of detail has important implications for anatomical segmentation. For instance, the visualization of transitions between CA1 and SUB, as well as presubicular islands, allows for a more precise delineation of subicular boundaries. Similarly, whereas the DG is often merged with CA4 [2, 70, 71], the present resolution enables separation of its granular cell and molecular layers. Finally, the ability to distinguish different strata within the SRLM opens new avenues to investigate layer-specific alterations. However, this level of laminar resolution was not consistently achieved across specimens and was therefore not formally quantified. Such approaches may be particularly relevant for layers that are typically less studied histologically due to their low neuronal density.

### 8.5. Limitations and Future Directions

Despite the unprecedented resolution and anatomical precision achieved in this study, several limitations should be acknowledged.

First, the sample size was relatively small (*N* = 19). Although the high resolution likely improved measurement precision and statistical power, allowing us to detect differences between Braak stages I and II, we cannot exclude that additional effects or regional differences may have been identified in a larger cohort. Notably, some pathology-thickness associations showed relatively large effect sizes but did not reach statistical significance, likely due to limited power.

Second, the cohort was not restricted to pure Alzheimer’s disease-related tau pathology. It included a mixture of Alzheimer’s disease-related tau and primary age-related tauopathy (PART), as well as co-pathologies such as ARTAG, AGD, LBD, and LATE-NC. In some individuals, tau pathology was not the primary driver of clinical symptoms, which were instead attributed to FTLD-TDP or LBD. Although we accounted for these (co)pathologies and conducted sensitivity analyses excluding FTLD-TDP, all TDP-43-positive, α-synuclein-positive, and ARTAG-positive cases, their presence may still have influenced the results. Furthermore, one individual presented concomitant tau, TDP-43, and α-synuclein pathology within the MTL. Such triple-positive pathology has been associated with greater MTL neurodegeneration than isolated proteinopathies[29], but its specific contribution could not be investigated because of the rarity of these cases and the limited sample size. More generally, the present cohort did not allow us to formally disentangle the independent or synergistic contributions of these co-pathologies to the MRI-visible atrophy pattern. Future studies including larger numbers of mixed-pathology cases should specifically investigate the impact of combined proteinopathies, particularly triple-positive cases, on regional MTL atrophy.

Third, findings related to TDP-43 should be interpreted with caution. The prevalence of TDP-43 pathology was relatively high compared with what would typically be expected in individuals at such early Braak NFT stages. This likely reflects both the inclusion of FTLD-TDP cases in addition to LATE-NC and the fact that our cohort consisted predominantly of very old cognitively unimpaired individuals, a population in which age-related TDP-43 pathology is particularly common. Moreover, analyses involving TDP-43 grouped heterogeneous conditions (e.g., different FTLD-TDP subtypes and LATE-NC), which differ in their regional TDP-43 distribution [51, 59, 60] and likely in TDP-43 species [51, 45, 86]. In addition, TDP-43 quantification was based exclusively on neuronal cytoplasmic (pre)inclusions and did not include dystrophic neurites. Thus, the specific contribution of TDP-43 pathology to the observed MRI alterations remains difficult to establish. Nevertheless, the persistence of the tau × TDP-43 interaction after excluding FTLD-TDP cases suggests that this interaction is not solely driven by FTLD-TDP and may also reflect the contribution of LATE-NC.

Fourth, although regions of interest were validated using neuropathology, and segmentation was improved by the high resolution, some anatomical boundaries remain challenging to delineate using MRI alone. For instance, CA2 and CA3 may not always be reliably distinguished, potentially leading to measurements being inadvertently taken in adjacent anatomical regions.

Finally, although our findings support the possibility of network-mediated effects of early tau pathology, we could not directly assess the potential contribution of other early-affected structures such as the amygdala. Future studies combining *ex vivo* MRI with quantitative analyses of amygdala subnuclei may help disentangle the relative contribution of entorhinal and amygdalar pathways to early hippocampal degeneration.

Future studies should include more specimens and aim to disentangle pathology-specific atrophy patterns (e.g., LATE-NC vs FTLD-TDP). The 50 μm in-plane resolution can also be used to examine laminar-specific effects of tau pathology, particularly in the CA1 SRLM. Additionally, more refined segmentation of BA35 and ERC subfields and layers could provide deeper insights into the earliest structural changes associated with tau aggregation.

### 8.6. Conclusion

This study demonstrates that ultra-high-resolution *ex vivo* MRI enables the visualization of previously inaccessible microstructural features of the MTL and provides a powerful tool to investigate early neurodegenerative changes. Our findings reveal that tau-related atrophy is detectable as early as Braak stage II, with a preferential involvement of anterior CA1 and SUB. These results provide new insights into the spatial and laminar specificity of early tau pathology and are compatible with the hypothesis of retrograde trans-synaptic propagation of tau-related neurotoxicity, although alternative network mechanisms involving other early-vulnerable regions cannot be excluded.

## Supplementary Material

Supplementary Files

This is a list of supplementary files associated with this preprint. Click to download.

- Salmanexvivomrisupplements22June2026.docx

## Figures and Tables

**Figure 1:**
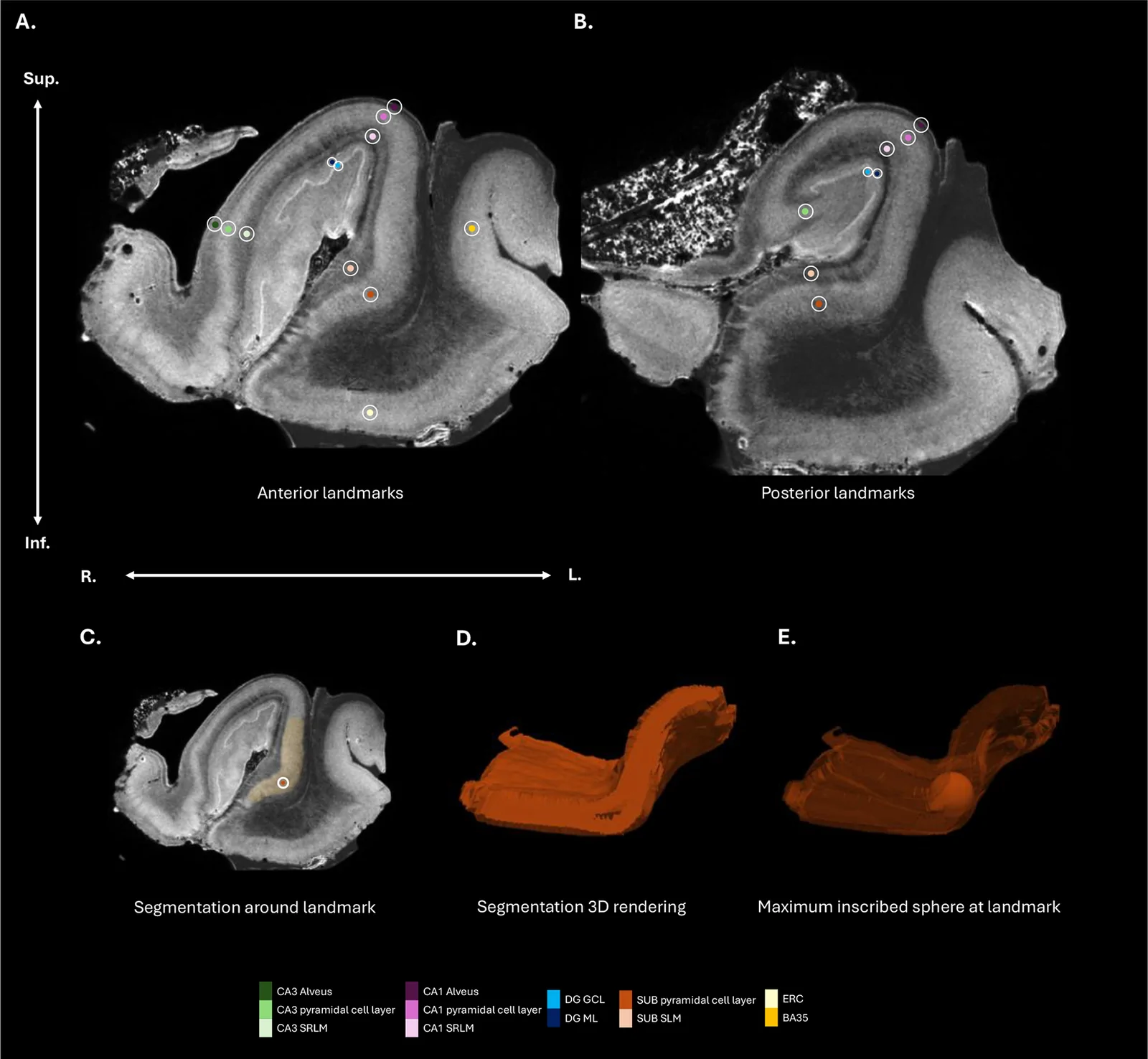
Cortical thickness measurement locations and processing pipeline. **(A–B)** Landmark locations for cortical subregions on **(A)** anterior and **(B)** posterior locations. For each landmark (dot), cortical thickness was measured using semi-automated active contour segmentation in ITK-SNAP (with possible manual correction) to segment a portion of the surrounding gray matter extending ~5 mm from the dot in all directions **(C–D).** Thickness at each location was defined as the diameter of the largest sphere overlapping the dot and fully contained within the gray matter segmentation (**E;** example shown anterior subiculum pyramidal cell layer). **Abbreviations**: BA35; Brodmann area 35; CA, Cornu Ammonis; DG, dentate gyrus; ERC, entorhinal cortex; GCL, granular cell layer; ML, molecular layer; SUB: subiculum S(R)LM, stratum (radiatum) lacunosum moleculare.

**Figure 2.**
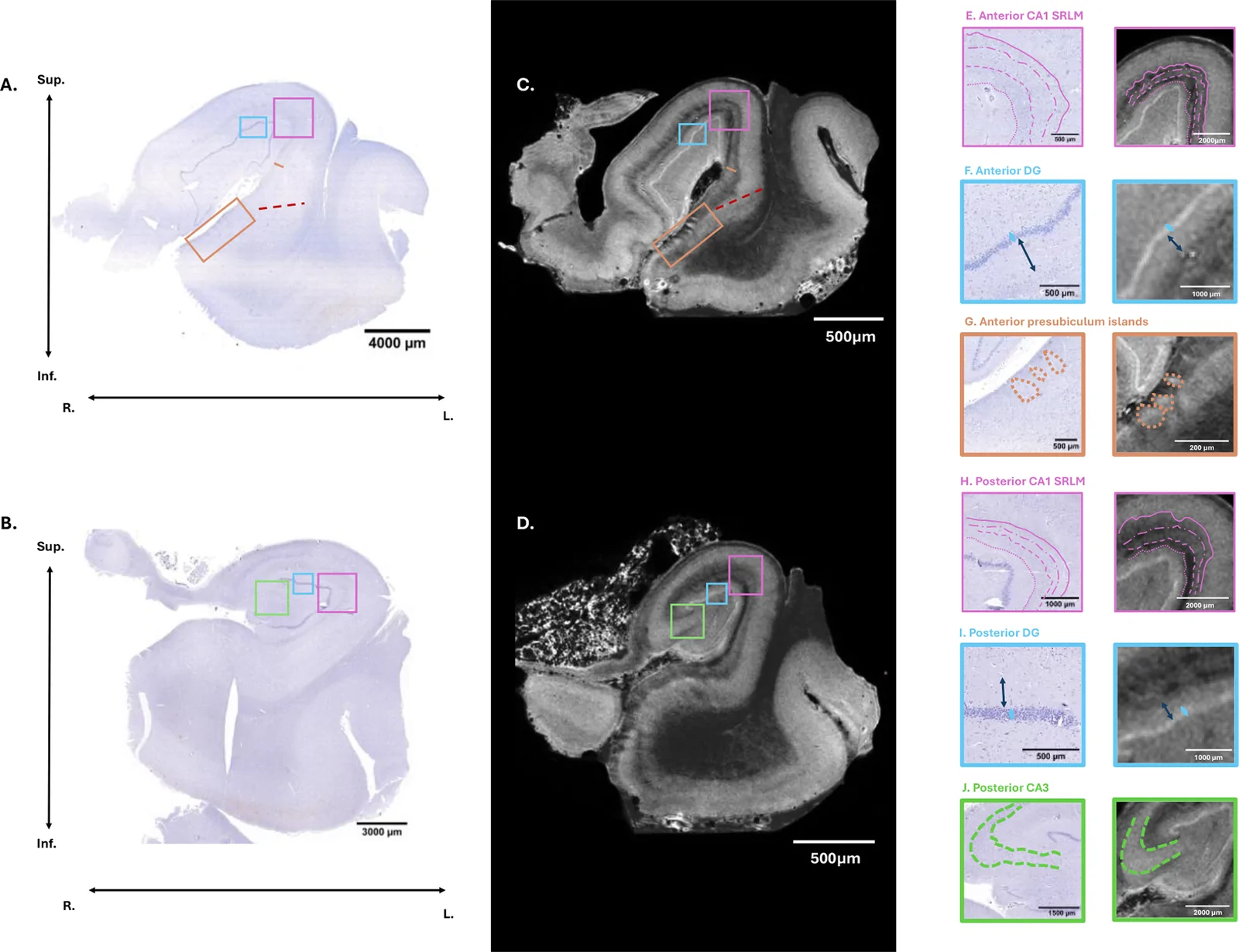
Multimodal comparison of medial temporal lobe (MTL) anatomy. **(A–B)** AT8-immunostained sections and **(C–D)** corresponding *ex vivo* MRI at 50 μm resolution. **(A, C)** Show the anterior MTL and **(B, D)** the posterior MTL. Blue boxes highlight the dentate gyrus (DG), where two layers can be distinguished (see **F, I**). The granule cell layer (GCL; light blue) appears as densely packed neurons in histology and as a thin hyperintense band in MRI. The molecular layer (ML; dark blue), located between the GCL and the stratum radiatum lacunosum moleculare (SRLM) of the cornu ammonis (CA), is relatively acellular and appears hypointense compared with the GCL, but hyperintense relative to the dark SRLM band. Pink boxes highlight the SRLM of CA1, which shows internal stratification in histology (see **F, H**). Three strata can be distinguished and appear as hypointense bands in MRI. The solid beige line marks the transition between the S(R)LM of CA1 and the subiculum (SUB), reflecting the loss of the stratum radiatum in SUB. The red dashed line (**A,B**) indicates the transition between the pyramidal cell layers of CA1 and SUB, seen in histology as a decrease in neuronal density and in MRI as reduced contrast. The beige boxes highlight the presubiculum (preSUB), characterized by clusters (“islands”) of neurons (dashed outlines), approximately 500 μm in size (see **G**). These appear in histology as small regions of lower neuronal density and as hyperintense structures in MRI. The green box shows the emergence of CA3 (see **J**, dashed lines), where pyramidal neurons organize into a well-defined band in histology, corresponding to a distinct hyperintense band emerging from CA4 in MRI. **Abbreviations:** CA, Cornu Ammonis; DG, dentate gyrus; PreSUB, presubiculum; SRLM, stratum radiatum lacunosum moleculare

**Figure 3:**
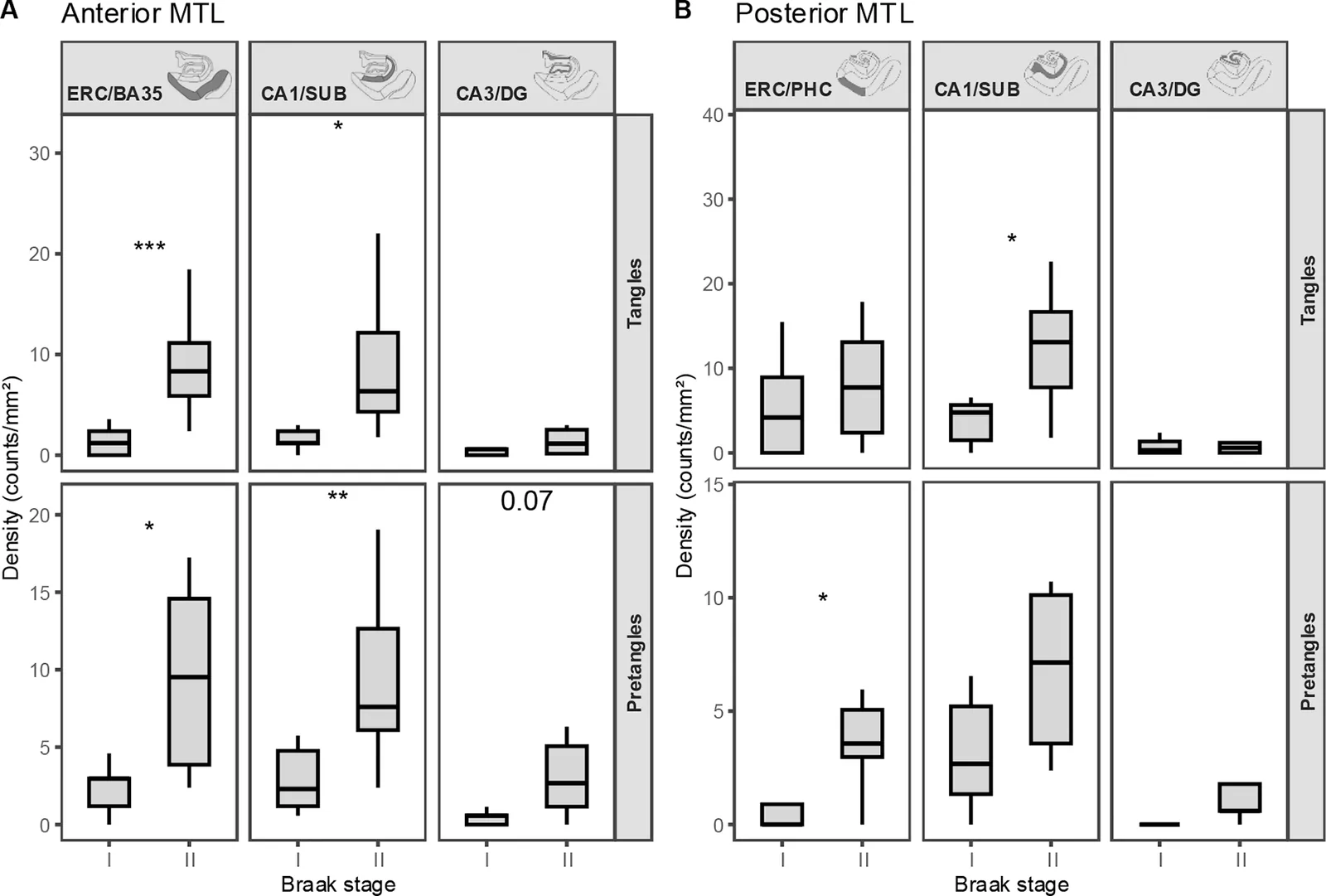
Distribution of tau pathology (pretangles and tangles) across MTL regions. Boxplots show the mean lesion density per mm^2^ for each group (Braak stage I or II) and the (A) anterior and (B) posterior MTL subregions. The regions where lesions were quantified are indicated graphically for each pannel. Statistical differences were assessed using rank-based ANOVA and are indicated as *P* < 0.05 (*) and *P* < 0.01 (**). **Abbreviations**: BA35; Brodmann area 35; CA, Cornu Ammonis; DG, dentate gyrus; ERC, entorhinal cortex; PHC: Parahippocampal cortex, SUB, Subiculum.

**Figure 4:**
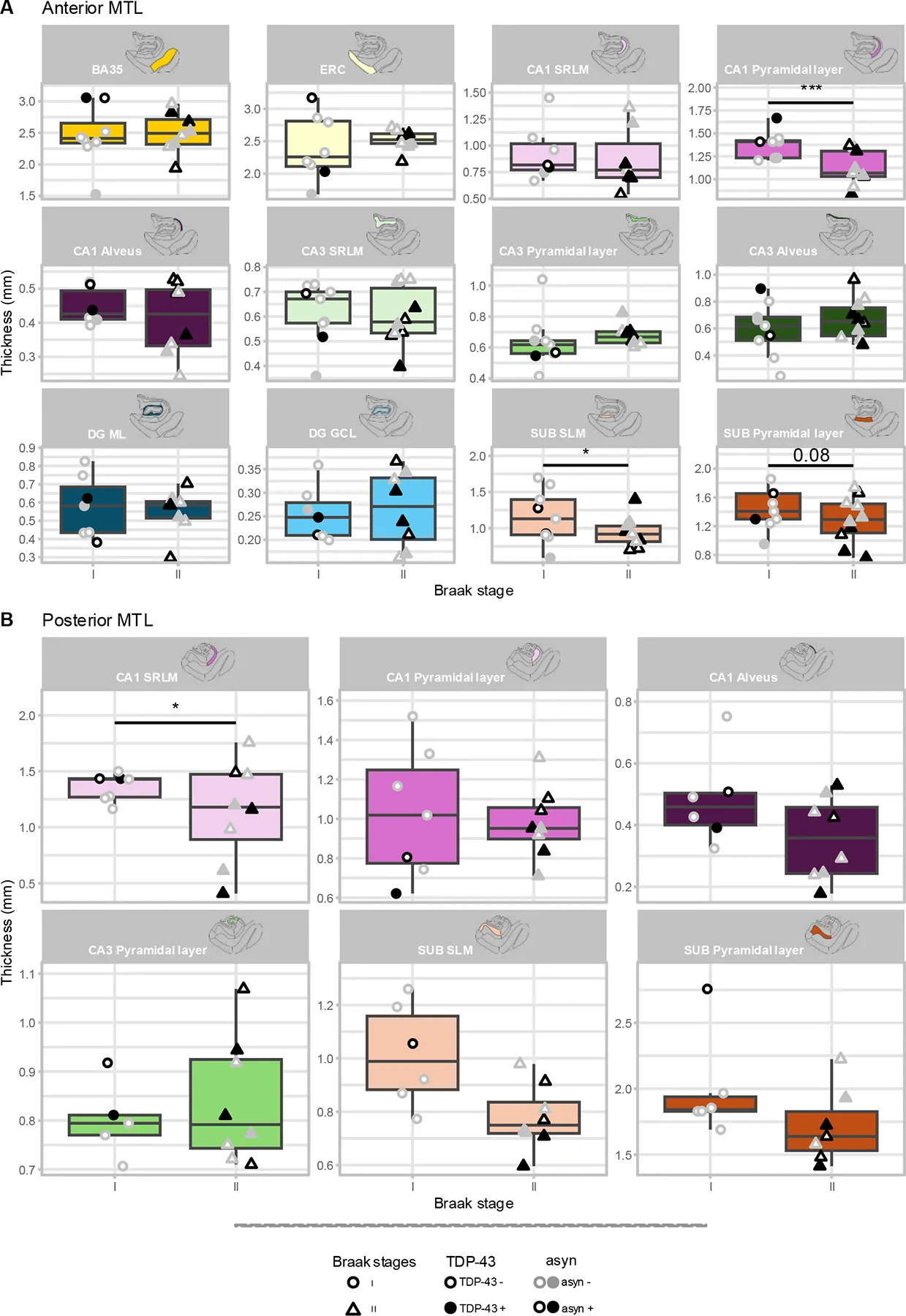
MTL thickness differences between Braak stage I and II individuals. Boxplots show the mean thickness ± standard error of the mean of each region for each group (Braak stage I or II) with individual raw data points overlaid in (**A**) anterior and (**B**) posterior MTL subregions. Colors represent the different regions where thickness was quantified and are also indicated graphically. Individual dots are coded by Braak stage (circles for stage I, triangles for stage II), by TDP-43 status (empty for negative, filled for positive), and by α-synuclein status (grey for negative, black for positive), see also figure legend. Statistical differences were assessed using rank-based ANCOVA, adjusted for age, sex, and presence of co-pathologies (α-synuclein, TDP-43, ARTAG; coded as binary variables), and are indicated as *P* < 0.05 (*), *P* < 0.01 (**), and *P* < 0.001 (***). Variance inflation factors (all VIF < 3) indicated no evidence of problematic multicollinearity among predictors. **Abbreviations**: BA35; Brodmann area 35; CA, Cornu Ammonis; DG, dentate gyrus; ERC, entorhinal cortex; GCL, granular cell layer; ML, molecular layer; SUB: subiculum S(R)LM, stratum (radiatum) lacunosum moleculare.

**Figure 5:**
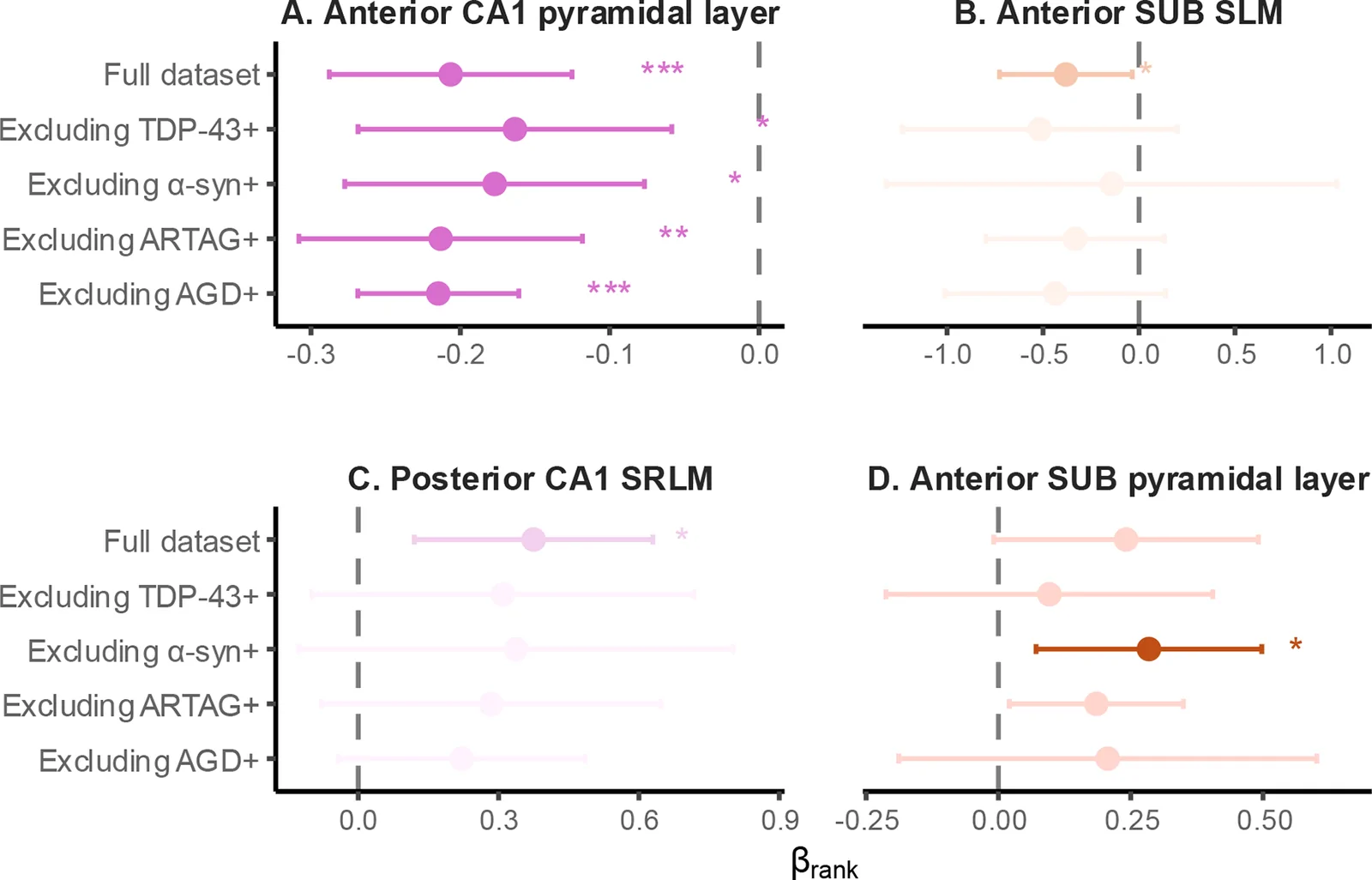
Sensitivity analyses of the association between Braak NFT stage and MTL thickness. Forest plots display rank-based ANCOVA estimates (*β_rank*) and approximate 95% confidence intervals for the effect of Braak NFT stage (II versus I) on thickness measures in (**A**) the anterior CA1 pyramidal layer, (**B**) the anterior subiculum SLM, (**C**) the posterior CA1 SRLM, and (**D**) the anterior subiculum pyramidal layer. Analyses were repeated in the full dataset and after excluding individuals with TDP-43 pathology, α-synuclein pathology, ARTAG, or AGD. Dark colors indicate statistically significant associations, whereas light colors indicate non-significant associations. Significance levels are indicated as *P* < 0.05 (*), *P* < 0.01 (**), and *P* < 0.001 (***). βrank estimates represent the adjusted effect of Braak stage II relative to Braak stage I. Negative βrank values indicate lower thickness associated with Braak stage II after adjustment for demographic factors and co-pathologies. Positive βrank values reflect the residual Braak effect after accounting for co-pathologies and should not be interpreted as evidence of greater thickness in Braak stage II individuals.. Abbreviations: AGD, argyrophilic grain disease; ARTAG, aging-related tau astrogliopathy; CA, Cornu Ammonis; NFT, neurofibrillary tangles; S(R)LM, stratum (radiatum) lacunosum moleculare; SUB, subiculum.

**Figure 6:**
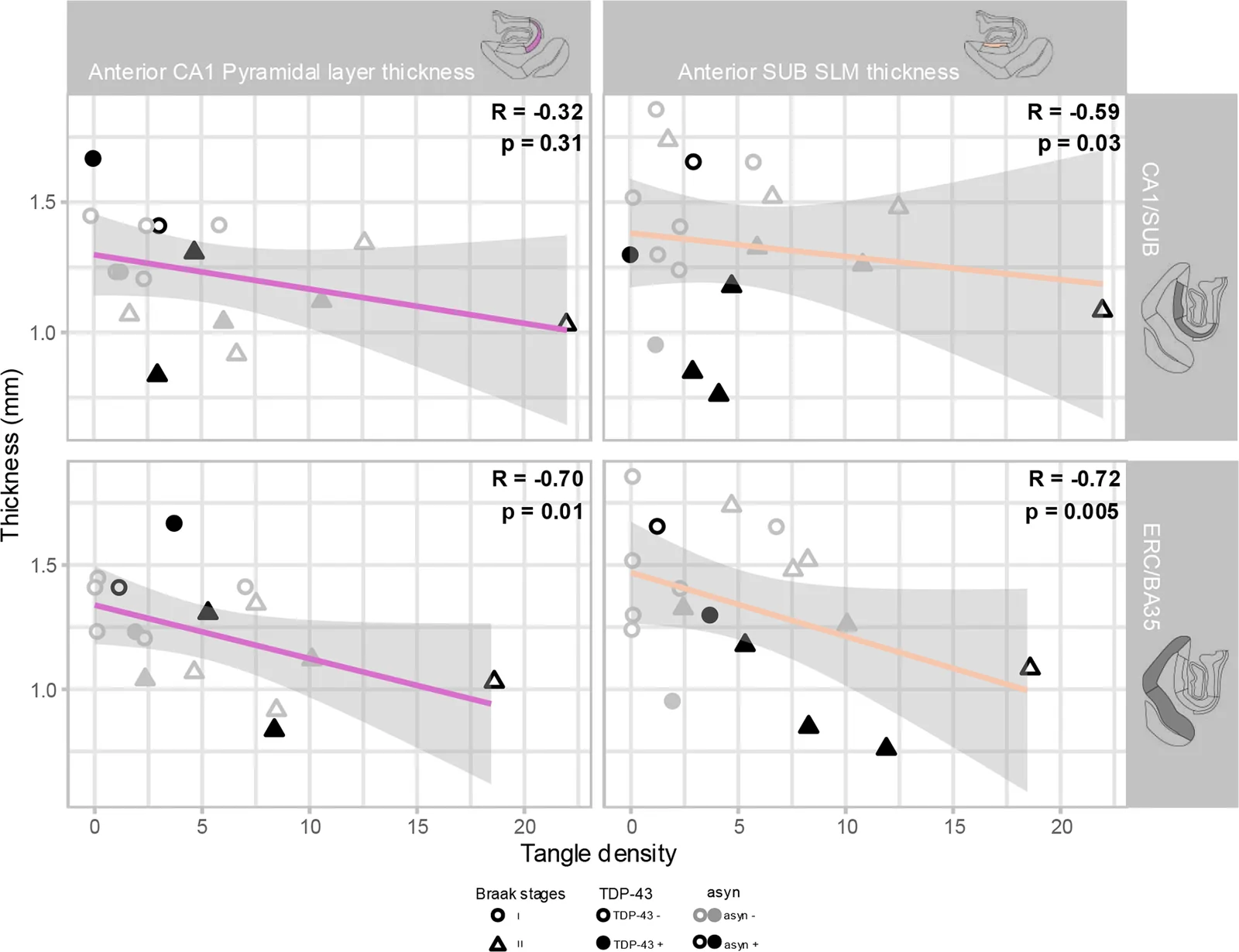
Correlations between MTL subregion thickness and NFT density in ERC/BA35 (Braak I region) and anterior CA1/SUB (Braak II region). Raw data are shown, while partial Spearman correlations were computed, adjusted for age, sex, and the presence of co-pathologies (TDP-43, α-synuclein and ARTAG). Colors indicate the different regions where thickness was measured and are also represented graphically in the column titles. The regions where NFT density was measured are displayed in grey as part of the raw titles. Individual data points are coded by Braak stage (circles for stage I, triangles for stage II), TDP-43 status (empty for negative, filled for positive), and α-synuclein status (grey for negative, black for positive). **Abbreviations**: BA35; Brodmann area 35; CA, Cornu Ammonis; ERC, entorhinal cortex; NFTs, neurofibrillary tangles; SLM, stratum lacunosum moleculare; SUB, subiculum.

**Figure 7:**
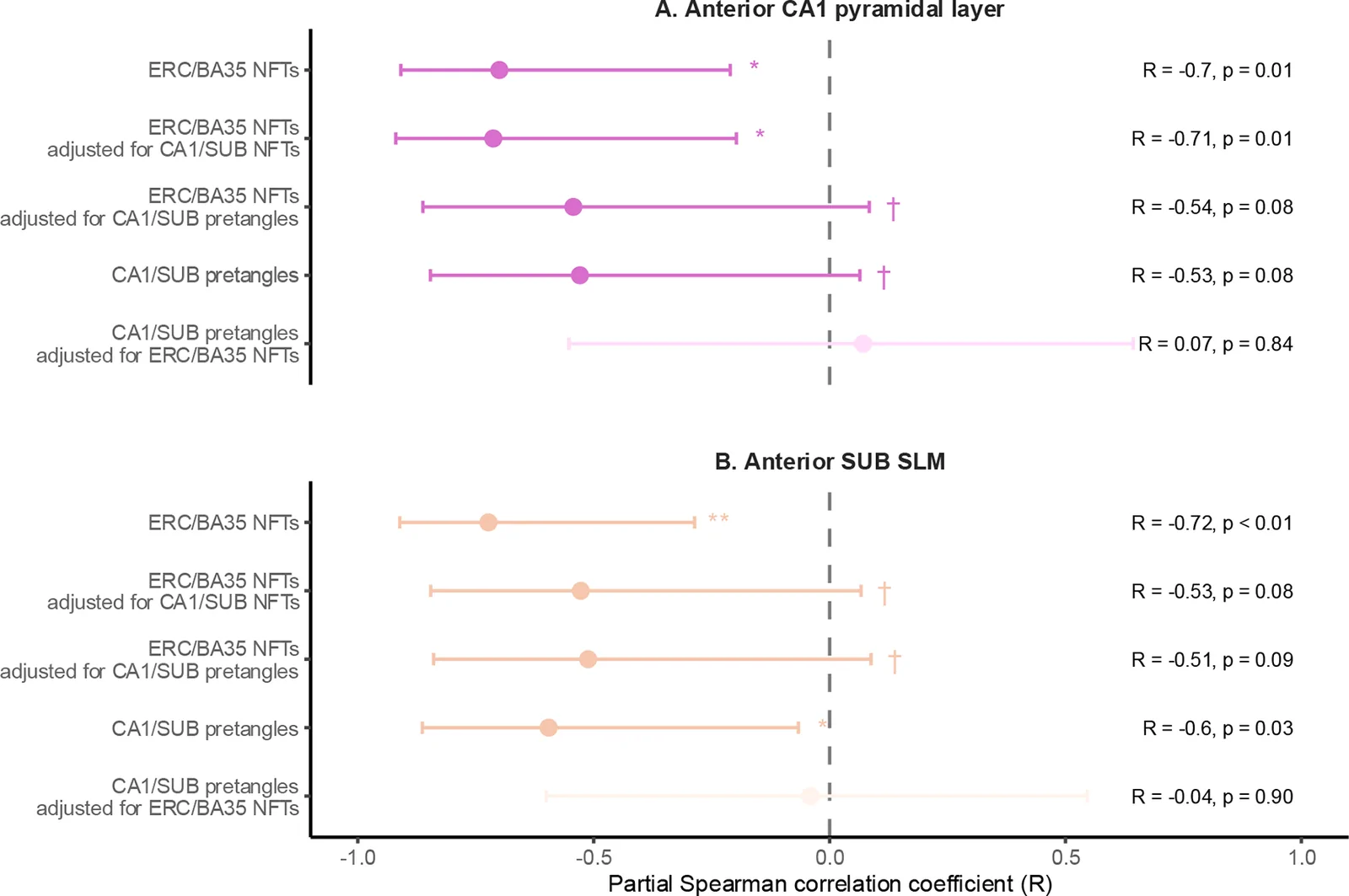
Evidence for a distant effect of (trans)entorhinal tau pathology on hippocampal thinning. Forest plots show partial Spearman correlation coefficients (R) and 95% confidence intervals between ERC/BA35 NFT burden and thickness of the (**A**) anterior CA1 pyramidal layer and (**B**) anterior subiculum SLM. Correlations are shown before adjustment, after adjustment for local NFT burden in CA1/SUB, and after adjustment for local pretangle burden in CA1/SUB. Corresponding analyses examining local pretangle burden are shown before and after adjustment for ERC/BA35 NFT burden. Dark colors indicate significant or trend-level associations (*P* < 0.10), whereas light colors indicate non-significant associations. Significance levels are indicated as *P* < 0.05 (*), *P* < 0.01 (**), and trend-level associations (†, *P* < 0.10). Abbreviations: BA35, Brodmann area 35; ERC, entorhinal cortex; NFT, neurofibrillary tangle; SLM, stratum lacunosum moleculare; SUB, subiculum.

**Table 1 :** Demographics.

	Braak I	Braak II	*p*
N	9	10	
Age at death (years)	80.9 ± 5.71	88.8 ± 7.20	0.03
Sex (% Male)	56%	80%	0.35
Cognitive status (% Cognitive Impairment)	22%	30%	0.99
Aβ phase	2.2 ± 1.8	1.7 ± 2.26	0.66
CERAD score	0.2 ± 0.44	0.2 ± 0.63	0.60
**Co-pathologies**			
TDP-43 (% of positive cases)	22%	50%	0.34
LATE-NC (% cases)	11%	40%	0.30
FTLD-TDP (% cases)	11%	10%	0.99
α-syn (% of positive cases)	22%	50%	0.35
ARTAG (% of positive cases)	0%	50%	0.03
AGD (% of positive cases)	11.11%	20%	0.99
Postmortem delay (hours)	39.67 ± 27.04	26.2 ± 13.80	0.15

**Abbreviations**: AGD, Argyrophilic Grain Disease; ARTAG, Aging-related tau astrogliopathy; CERAD, Consortium to Establish a Registry for Alzheimer’s Disease; LATE-NC, Limbic-predominant Age-related TDP-43 Encephalopathy neuropathological changes; FTLD-TDP, frontotemporal lobe degeneration caused by TDP-43 pathology.
